# Characteristics of* Salmonella* spp. Isolated from Wild Birds Confiscated in Illegal Trade Markets, Rio de Janeiro, Brazil

**DOI:** 10.1155/2016/3416864

**Published:** 2016-01-11

**Authors:** Carlos Alexandre Rey Matias, Ingrid Annes Pereira, Maiara dos Santos de Araújo, André Felipe Mercês Santos, Rudi Pereira Lopes, Sandra Christakis, Dália dos Prazeres Rodrigues, Salvatore Siciliano

**Affiliations:** ^1^Departamento de Epidemiologia e Saúde Pública, Instituto de Veterinária, Universidade Federal Rural do Rio de Janeiro, 23890-000 Seropédica, RJ, Brazil; ^2^Escola Nacional de Saúde Pública Sérgio Arouca, Fundação Oswaldo Cruz, 21041-210 Rio de Janeiro, RJ, Brazil; ^3^Laboratório de Referência Nacional de Enteroinfecções Bacterianas, Instituto Oswaldo Cruz, 21040-360 Rio de Janeiro, RJ, Brazil; ^4^Laboratório Central de Saúde Pública de Santa Catarina, 88010-002 Florianópolis, SC, Brazil

## Abstract

The prevalence of* Salmonella* spp. was investigated in 109 wild birds poached in the illegal wildlife trade in Rio de Janeiro; most of them are passerines from Thraupidae family and three from Psittacidae. One strain of* Salmonella* ser. Typhimurium and two strains of* Salmonella* ser. Panama were isolated from passerine species and all of them showed resistance to multiple antimicrobial drugs, like ampicillin, ceftriaxone, ceftiofur, tetracycline, gentamicin, nalidixic acid, ciprofloxacin, and enrofloxacin. PFGE showed 100% similarity among the* Salmonella* ser. Typhimurium strain isolated from a Temminck's seedeater (*Sporophila falcirostris*) and the strains isolated from a human outbreak, in southern Brazil. The two* Salmonella* ser. Panama strains isolated from two chestnut-capped blackbirds (*Chrysomus ruficapillus*) present in the same catch showed the same clonal origin and have never been associated with epizooties and human outbreaks. Potential for dissemination of resistant* Salmonella* through situations offered by captive management and the isolation of the same strain from wild birds and human sources may become a problem for the conservation of natural populations and to public health.

## 1. Introduction

Brazil is one of the richest countries in the world in terms of biodiversity, harboring an estimated 10 to 12% of all known species including more than 1800 bird species, of which some are endemic and a number are considered threatened [1, 2].

Illegal wildlife trade is a lucrative activity, considered the third largest illegal trade in the world, behind arms trafficking and illicit drug trade [3]. The Brazilian law considers the capture of wild animals and their maintenance in captivity without legal permission criminal.

After habitat loss, wildlife poaching and hunting are considered the most important causes of population declines and could significantly affect ecosystem dynamics [4]. Besides these consequences, risk of disease dissemination has to be considered, given that captivity allows greater contact between species, favoring the transmission of infectious agents [1, 2, 5], and provides disease transmission mechanisms that can not only cause human disease outbreaks, but also threaten livestock, native wildlife populations, and ecosystem health [6].

Wild birds and migratory species may act as vectors in the transmission of different exotic microorganisms and may have a role in the spreading of emerging and reemerging pathogens [7, 8]. They can carry a wide range of zoonotic pathogens, including enteric pathogenic bacteria, either being themselves diseased or being seemingly healthy carriers or hosts of infected vectors [9].

Bacteria of the* Salmonella* genus colonize the digestive tract of reptiles, birds, and mammals, including humans, and are involved in gastroenteritis and other kinds of infections [10]. Salmonellosis causes gastroenteritis in humans in both developed and developing countries, leading to economic losses and animal and human illnesses, being the second most often reported zoonotic disease and the most important bacterial food-borne disease in industrialized countries [11]. Commonly found in the intestinal tract of wild birds [7], this microorganism may be a source of infection for humans and domestic animals, especially* Salmonella* ser. Typhimurium, which has a wide host range and can be associated with disease in humans, livestock, waterfowl, rodents, and birds [12, 13]. Using wild birds as sentinels for this food-borne pathogen also allows for the evaluation of its role in the spread of antimicrobial resistance in the environment, a worldwide emergency problem.

The goals of the present study were to investigate the prevalence of* Salmonella* species in cloacal swab samples of wild birds that were pouched in the illegal trade in Rio de Janeiro, Brazil, to evaluate their antimicrobial resistance profiles, and, using a subtyping method, to evaluate the spread and transmission of resistant strains to the environment and to humans, detecting genetic markers for the main antimicrobials applied to* Salmonella* infections used in veterinary practices.

## 2. Materials and Methods

Wild birds were confiscated in illegal trade markets by the police in Rio de Janeiro, Brazil, from March 2011 to March 2012 and sent to the Rehabilitation Center of Wild Animals (CETAS). The cloacae samples were obtained from birds chosen randomly in a total of nine apprehensions. One hundred and nine birds, representing 30 species, were chosen according to their diversity ratio in each apprehension, with the highest frequency being the Thraupidae family. Regarding all species, each species was represented by a maximum of sixteen and a minimum of nine birds. Samples were obtained by clinical procedures, using swabs introduced into Cary Blair media under refrigerated conditions and taken to the National Reference Laboratory of Intestinal Bacterial Infections (LABENT), at the Oswaldo Cruz Institute (FIOCRUZ), Rio de Janeiro, Brazil, in order to conduct the microbiological assays.

The collected material was transferred to Nutrient Broth (Difco) (37°C/18–24 hours). Subsequently, the samples were enriched in Rappaport-Vassiliadis Broth (42°C overnight), Silliker Medium, and Muller-Kauffmann Medium and incubated overnight at 37°C and then isolated on Hektoen enteric agar (OXOID) (37°C/18–24 hours). Suspected colonies were confirmed by using Triple Sugar Iron (Difco) and then biochemically characterized through susceptibility to L-lysine decarboxylase and to citrate as a carbon source and through mobility and production of hydrogen sulfide and indole by SIM medium. The identification of subspecies was determined using substrates according to Grimont and Weill [14]. The antigenic characterization, to identify the surface antigens with somatic antisera and flagella antigens with flagellar antisera, followed the Kauffmann-White Scheme. The antigenic characterization was performed by slide agglutination with somatic and flagellar poly- and monovalent antisera and prepared at the LABENT, the Oswaldo Cruz Institute (FIOCRUZ), Rio de Janeiro, Brazil. The identification of the specific serovar was performed and represented according to the criteria reported by Grimont and Weill [14].

Susceptibility testing was performed by the Minimum Inhibitory Concentration Assay (MIC) in Agar and Broth to determine the lowest concentrations of different antimicrobial drugs. Each one was evaluated in a serial dilution according to the protocol by the Clinical and Laboratory Standards Institute [15] with ampicillin, ceftriaxone, ceftiofur, tetracycline, trimethoprim/sulfamethoxazole 19 : 1, chloramphenicol, gentamicin, nalidixic acid, ciprofloxacin, enrofloxacin, and nitrofurantoin. The following reference strains were used for quality control of the antimicrobial susceptibility test:* Staphylococcus aureus* ATCC25923,* Pseudomonas aeruginosa* ATCC27853, and* Escherichia coli* ATCC25922.

The antimicrobial resistance genes were determined by a PCR assay. At this stage, strains resistant to 2nd- and 3rd-generation cephalosporins and last generation quinolones were primarily selected. DNA extraction and quantification were conducted using a Qiagen kit. The sequences of forward and reverse primers used as indicators for the detection of gene cassettes encoding resistance were those described by Pitout et al. [16] for quinolones and Olesen et al. [17] for *β*-lactamases. These primers sets generated amplicons of 516 pb, 469 pb, 417 pb, and 320 pb for PMQR; 920 pb for *bla*_cmy_; 250 pb for* aac(*3′*)IIa*; 482 pb for* aac(*6′*)IB*; 250 pb for Integrase; 700 pb for Integron class I, and 593 pb for *bla*_CTXm_ [18].

PFGE for molecular subtyping was performed according to the PULSENET protocol using a CHEF DRIII and* Salmonella* ser. Braenderup H9812 was used as the reference strain. Electrophoresis conditions were an initial switch time of 2.16 sec, a final switch time of 63.8 sec, and a run time of 21 h. The analysis and comparison of PFGE patterns were performed using the BioNumerics Software [19].

## 3. Results

**Figure 1:**
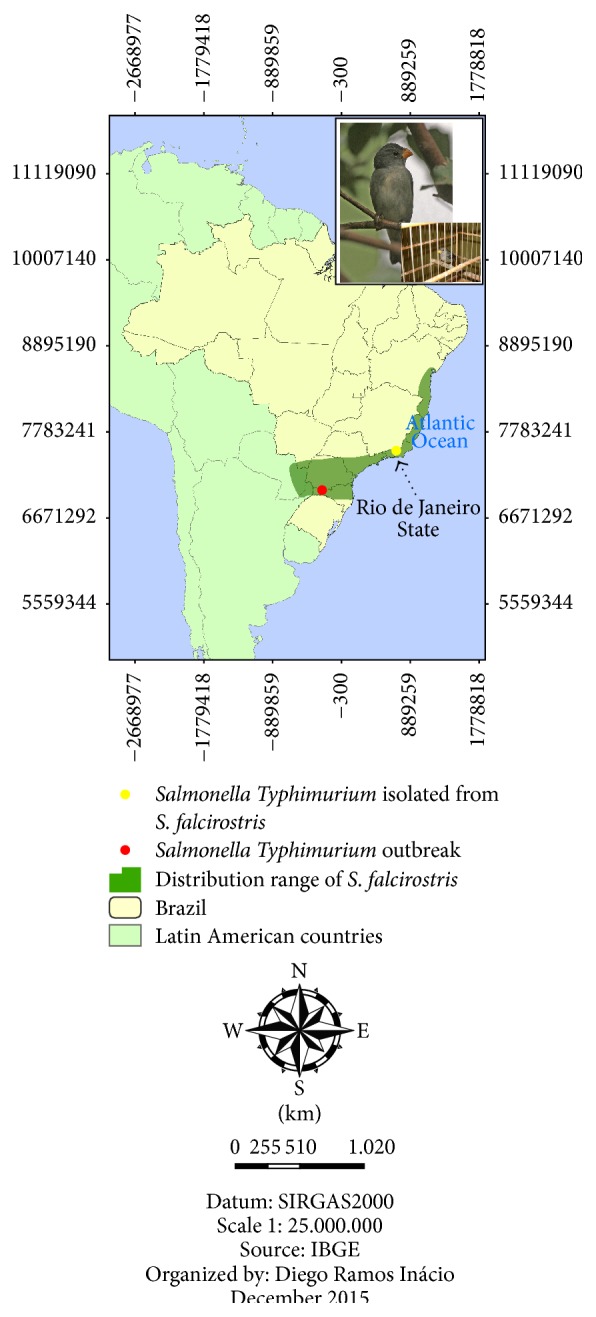
Distribution range of Temminck's seedeater (*Sporophila falcirostris*) and* Salmonella* isolation location in a bird and humans.

**Figure 2:**
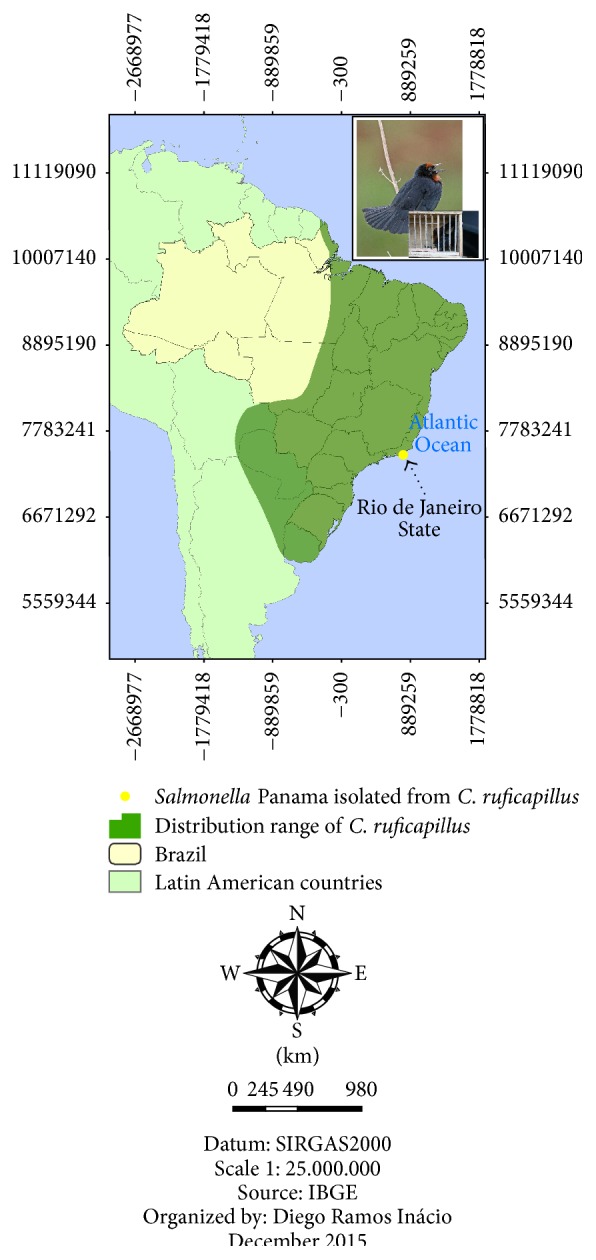
Distribution range of the chestnut-capped blackbird (*Chrysomus ruficapillus*) and* Salmonella* isolation location in two birds.

*Salmonella* spp. were isolated from three samples, yielding an isolation rate of 2.75% regardless of bird species.* Salmonella* ser. Typhimurium (O:4,5,12:i:1,2) was isolated from Temminck's seedeater (*Sporophila falcirostris*) (Figure 1) and, in a different apprehension,* Salmonella* ser. Panama (O:9,12:l,v:1,5) was isolated from two chestnut-capped blackbird (*Chrysomus ruficapillus*) specimens that were kept together in the same cage (Figure 2). All the birds are passerines and had no symptoms of disease.

Multidrug resistance was found in all three* Salmonella* isolates, with resistance ranging from 3 to 8 antimicrobial drugs (Table 1). All strains were susceptible to trimethoprim-sulfamethoxazole and chloramphenicol and resistant to ceftriaxone and ceftiofur. Among the two* Salmonella* ser. Panama strains, one showed resistance to ampicillin, ceftriaxone, ceftiofur, tetracycline, chloramphenicol, gentamicin, nalidixic acid, ciprofloxacin, and enrofloxacin and contained* aac(*3′*)IIa* gene, while antimicrobial resistance genes were not detected in the other.

**Figure 3:**
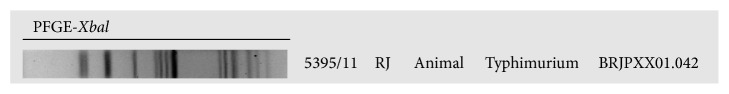
Pulsed-field gel electrophoresis profile showing the four* Xbal* patterns of the* Salmonella* serovar Typhimurium strain identified from a Temminck's seedeater fecal sample.

**Figure 4:**
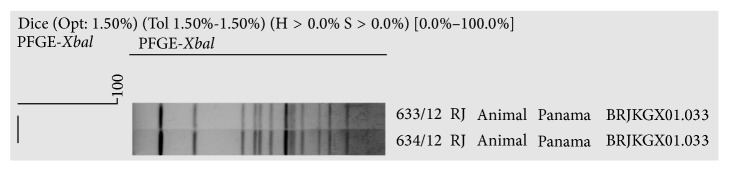
Pulsed-field gel electrophoresis profiles showing the four* Xbal* patterns of the two* Salmonella* serovar Panama strains identified from two chestnut-capped blackbird fecal samples.

The analysis and comparison of PFGE patterns of the isolated* Salmonella* ser. Typhimurium strain with the National Databank in Brazil showed 100% similarity with two strains from human sources, isolated later in southern Brazil, indicating that the strain is already circulating in the country (BRJPXX01.042) (Figure 3). Despite displaying different resistance profiles, the two* Salmonella* ser. Panama strains showed the same clonal origin, indicating that the two birds have a common source of infection. When compared to the database isolates from different sources, no common ancestry was found (Figure 4).

## 4. Discussion

The prevalence of* Salmonella* spp. among the samples evaluated from apparently healthy wild birds was low when compared to studies from dead or dying specimens [9, 20]. Despite the low detection rate (2.75%), these results are a sign that the isolated serovars circulate in the bird population. Although* Salmonella* spp. were isolated from three different wild birds, evidence of transmission to humans from wild birds is not generally established, but it has been shown that contact with other animals and their products are important in the process of human infection. All wild birds potentially carry human pathogens and, thus, handling these birds involves a risk to human health if good hygiene is not practiced. The characterized serovars* Salmonella* ser. Typhimurium and* Salmonella* ser. Panama circulate in Brazil and in other countries and can be isolated from human and animal sources [21].

Birds, especially passerines, are the main victims of illegal wildlife trade in Brazil [22]. The isolation of* Salmonella* in apparently healthy birds reinforces the needs for a monitoring program to predict epizootic events and to detect human outbreaks. Temminck's seedeater (*Sporophila falcirostris*) is an endemic species of the Atlantic Rain Forest that inhabits higher altitudes and has a granivorous feeding habit. The characterized serovar* Salmonella* ser. Typhimurium has been frequently detected in outbreaks that have affected men and livestock, especially poultry, since the 1990s [23]. The chestnut-capped blackbird (*Chrysomus ruficapillus*) is a species widely distributed throughout Brazil, inhabiting grasslands and flooded areas and feeding on insects, arthropods, small vertebrates, and fruits. The Panama serovar is not usually isolated in epizooties and human outbreaks in Brazil [23].

The Typhimurium serovar is frequently associated with disease in many different mammalian and avian host species [12]. Outbreaks of* Salmonella* ser. Typhimurium in humans having contact with wild passerine birds as a common source have been described in Norway causing the death of several birds [24], as well as New Zealand [25]. In this case, contact with dead birds, sick people, and the ingestion of contaminated food were responsible for the infection. Salmonellosis epizooties by* Salmonella* ser. Typhimurium were identified in birds from Norway between 1999 and 2000 [26] and, since 1973,* Salmonella* ser. Typhimurium has been isolated from dead passerines in the US [27]. In the latter, a study conducted by Hall and Saito [28] showed that this same serovar was primarily responsible for mortality events in wild birds between 1985 and 2004 and that the sources of infection were fecal-oral, social, and feeding behavior. Between 1995 and 2008, several cases of salmonellosis by* Salmonella* ser. Typhimurium were diagnosed in birds from Scotland [29]. Other outbreaks have also been reported in Canada, New Zealand, Sweden, and the United Kingdom, and birds can be a source of* Salmonella* infection to other animal species, including humans [20, 30, 31]. All these outbreaks occurred in temperate countries, in feeding places, where birds could be infected by fecal-oral transmission. In Brazil, people do not have the habit of feeding birds, and these animals decompose rapidly in tropical conditions. Moreover, several other countries like the US, Norway, and Sweden have better disease control programs than Brazil, demonstrating greater ability to detect epizootic events.

The results of antimicrobial susceptibility in the present study indicate that all isolates were multiresistant.* Salmonella* ser. Typhimurium evidenced that the resistance problem has remained almost unaltered during all the considered decades. In Brazil, the presence of antimicrobial resistance began in the 70s, with the prevalence of multidrug resistant PT193 in Recife, and the problem has continued virtually unaltered until today. Since then, the predominance of isolates, accompanied by antimicrobial traits, has been detected over the decades in samples isolated from human, animal, and food sources [32–34]. Although the resistance to antimicrobials in* Salmonella* serovars is an ecological phenomenon, it arises, mainly, from the natural competition among microorganisms. One study [35], which analyzed human enterobacteria isolates from 1920 and from African wild animals, showed low resistance to the antimicrobials, but, nevertheless, the presence of plasmids that transmit resistance factors was detected.

It must be noted that all the isolates were resistant to ceftiofur and ceftriaxone, 3rd-generation cephalosporins, the former used in veterinary medicine and the latter used to treat severe human* Salmonella* infections. The enrofloxacin is a quinolone exclusive for veterinary use. Although this quinolone is structurally similar to ciprofloxacin, a 2nd generation of fluoroquinolone, the literature showed different results related to their resistance profiles. The resistance to quinolones in* Salmonella* spp. is a warning to the scientific community with knowledge about the possibility of transmission of resistance by mechanisms mutations in target genes and/or by determinants in plasmids and transposons. The* aac(*3′*)IIa* isolated in one of the* Salmonella* ser. Panama strains mediates resistance to gentamicin. Despite the detection of phenotypic resistance to multiple antibiotics, most different serotypes isolated from clinical cases are resistant to various antimicrobials and carry the class 1 Integron gene, involved in antimicrobial multiresistance [36]. This gene was not observed in any samples analyzed by PCR. It is possible that other genes or mechanisms may be involved in the multiresistance to antimicrobials observed in the* Salmonella* strains identified in the present study.

According to Hilbert et al. [11], wild birds can acquire and disseminate* Salmonella* infections, including resistant strains, by direct contact with food-producing animals and with species that can act as vectors, such as insects, rodents, and other birds. These birds feed in areas potentially contaminated by human waste and can also be in direct contact with human activities, such as contaminated food and human waste. The level of infection in wild birds may simply reflect the level of intestinal carriage by humans. In these cases, the birds may act as reservoirs for resistant bacterial pathogens. These resistance profiles raise an alert for the need and importance of a surveillance program to prevent impacts on public health.

The epidemiological investigation of* Salmonella* spp. using molecular based methods is especially valuable. PFGE has been widely used to determine strain relatedness, confirm outbreaks, and identify the sources of the identified strains [19]. The illegal wildlife trade in Brazil can be the source of human salmonellosis outbreaks and can facilitate the dissemination of resistant* Salmonella* through situations offered by captive management, such as maintaining birds in overcrowded cages and offering contaminated food, which may become a problem for the conservation of natural populations and to public health. In the present study, PFGE was used for the subtyping of one* Salmonella* ser. Typhimurium strain and two* Salmonella* ser. Panama strains. The* Salmonella* ser. Panama has been isolated from many foods, animals, water, and patients presenting clinical cases of infection. This serovar is one of several serotypes that tend to cause more invasive disease than other serotypes and has been associated with bacteremia and meningitis, mainly in children. In Brazil its prevalence is low and is detected in northeast and southeast region. The PFGE analysis does not show similarity with strains isolated from different sources in Brazil. Results are similar to those obtained from a commercial salami processing line, which detected the presence of multiple PFGE profiles [37]. The PFGE analysis indicated that the serovar Typhimurium isolate is indistinguishable and/or highly related with two strains isolated from a food-borne disease outbreak in south Brazil. This result showed the relevant role of wild birds in public health to spread a Salmonella clone related with a food-borne outbreak. Less direct evidence has been reported by Heir et al. [38], regarding bird-to-human transmission, which used PFGE to analyze human Typhimurium strains in Norway. They found that the strains characteristic of wild birds accounted for 32% of sporadic human cases.

## 5. Conclusions

These results point to the relevance of curbing the trafficking of wild animals, which can be a source of salmonellosis and be responsible for animal and human outbreaks. Thus, it is recommended that integration policies should be implemented between the various bodies and institutions involved in combating wildlife illegal trade—including the environmental agencies of the various levels of the Brazilian government—and the entities responsible for health surveillance. This includes the implementation of the following measures: long-term actions to combat wildlife illegal trade and its withdrawal from nature; clarifying for the public the health risks of acquiring illegally sourced specimens; continuous monitoring of birds victims of wildlife illegal trade for the presence of* Salmonella*, by carrying out quarantine procedures to prevent the spread of strains with zoonotic potential and/or multiprofile bacterial resistance in different environments and for humans due to the release and disposal of these animals; and monitoring professionals involved in the management of wild species, since the handling of these animals exposes them to greater contact with zoonotic agents and microorganisms involved in nosocomial infections.

## Figures and Tables

**Table 1:** *Salmonella* isolated from wild birds in CETAS and tested for antibiotic resistance.

*Salmonella* serovar	Host species	Antimicrobial resistance^*∗*^										
		AMP	CRO	CEF	TCY	SXT	CHL	GEN	NAL	CIP	ENR	NIT
Typhimurium	Temminck's seedeater	I	R	R	R	S	S	S	R	S	R	I
Panama	Chestnut-capped blackbird	R	R	R	R	S	S	R	R	R	R	I
Panama	Chestnut-capped blackbird	S	R	R	S	S	S	R	S	S	I	I

^*∗*^AMP = ampicillin; CRO = ceftriaxone; CEF = ceftiofur; TCY = tetracycline; SXT = trimethoprim/sulfamethoxazole; CHL = chloramphenicol; GEN = gentamicin; NAL = nalidixic acid; CIP = ciprofloxacin; ENR = enrofloxacin; NIT = nitrofurantoin.

S = susceptible; I = intermediate; R = resistant.
